# Design and Implementation of a Multilevel Intervention to Reduce Hepatitis C Transmission Among Men Who Have Sex With Men in Amsterdam: Co-Creation and Usability Study

**DOI:** 10.2196/19100

**Published:** 2020-09-11

**Authors:** Tamara Prinsenberg, Paul Zantkuijl, Wim Zuilhof, Udi Davidovich, Janke Schinkel, Maria Prins, Marc van der Valk

**Affiliations:** 1 Department of Infectious Diseases Research and Prevention Public Health Service of Amsterdam Amsterdam Netherlands; 2 Department of Infectious Diseases Amsterdam Universitair Medische Centra University of Amsterdam Amsterdam Netherlands; 3 Amsterdam Infection & Immunity Institute Amsterdam Universitair Medische Centra University of Amsterdam Amsterdam Netherlands; 4 Soa Aids Nederland Amsterdam Netherlands; 5 Department of Social Psychology University of Amsterdam Amsterdam Netherlands; 6 Department of Medical Microbiology, Section of Clinical Virology Amsterdam Universitair Medische Centra University of Amsterdam Amsterdam Netherlands

**Keywords:** co-creation, mHealth, intervention, hepatitis C, prevention, risk reduction, MSM, HCV

## Abstract

**Background:**

In the Netherlands, transmission of hepatitis C virus (HCV) occurs primarily among men who have sex with men (MSM). Early HCV testing of at-risk MSM and immediate initiation of treatment will prevent onward transmission, but this may not be sufficient to eliminate HCV in a population with ongoing risk behaviors. Therefore, targeted socioculturally acceptable preventive measures, including behavioral interventions, are urgently needed. Currently, little contextually appropriate information about HCV or risk reduction interventions is available.

**Objective:**

The objective of this project was to develop an intervention to reduce HCV transmission among MSM in Amsterdam through a co-creation process, with the input of men from the targeted community directly impacting intervention content, design, and implementation.

**Methods:**

We developed a multilevel intervention targeting 6 levels: individual, community, professional, context, patient, and network. The intervention was developed in close cooperation between health professionals, gay community members, commercial stakeholders, and stakeholders from within the gay community. The co-creation process had 4 phases: a needs assessment, stakeholder engagement, co-creation, and implementation. The co-creation phase continued until consensus was reached between the researchers and community members on the intervention content and design. The final intervention, NoMoreC, was completed within 2 years, and implementation started in February 2018.

**Results:**

NoMoreC includes web-based and face-to-face components as well as an anonymous HCV testing service. The NoMoreC website provides information about hepatitis C, HCV transmission routes, risk reduction strategies, testing and treatment options, and partner notification. The face-to-face component comprises a risk reduction toolbox, training for health professionals, and providing tailored advice to sex on premises venues. NoMoreC is promoted by an active voluntary campaign team.

**Conclusions:**

Involving the community and stakeholders in the creation of NoMoreC has been the main strength of this project. It has resulted in an intervention with various components that resonates with the gay community at risk of HCV infection. The uptake and acceptability of the described intervention will be evaluated in the future. The description of the co-creation process and implementation of the project may serve as a rich and useful source for others who want to develop culturally and context appropriate HCV interventions.

## Introduction

Hepatitis C virus (HCV) infection is a major public health problem: An estimated 71 million people worldwide are living with chronic HCV infection, which, if left untreated, may progress to serious liver disease [1]. In 2016, approximately 399,000 people died from HCV-related cirrhosis and liver cancer, and the number of deaths increases each year [2]. In the Netherlands, HCV transmission occurs primarily among HIV-positive men who have sex with men (MSM), as HCV incidence dropped to nearly zero among people who inject drugs [3-5]. Since 2000, there has been an unexpected and substantial increase in HCV incidence among HIV- positive MSM globally [6,7]. Data from the international CASCADE collaboration demonstrated a significant overall increase in HCV incidence among HIV-positive MSM — from 0.07/100 person-years in 1990 to 1.8/100 person-years in 2017 [8]. The incidence of HCV reinfection among HIV-positive MSM is 3-10 times higher than the primary infection incidence, and more recent data show ongoing HCV transmission among HIV-negative MSM using pre-exposure prophylaxis [9-14].

Recent improvements in HCV therapy have resulted in multiple highly tolerable direct-acting antiviral (DAA) regimens with cure rates of over 95%. [15] In many countries, DAA therapy is available for patients with chronic HCV [16]. In the Netherlands, unrestricted access to DAA for all chronically HCV-infected individuals has been available since 2015. The uptake of HCV treatment in HIV/HCV co-infected individuals in the Netherlands has been very rapid: 15 months after the introduction of DAA, 83% of HIV/HCV co-infected MSM were cured [17].

Early testing of at-risk MSM and immediate initiation of treatment prevent onward transmission, but this may not be sufficient to eliminate HCV in a population with ongoing risk behavior [18-20]. Uptake of DAAs differs greatly between countries [21], and international HCV transmission in HIV-positive MSM persists [22,23]. Salazar-Vizcaya et al [18] showed that prevention of high-risk behavior alone could result in a considerable reduction of HCV transmission among HIV-infected MSM based on different modeled scenarios on hypothetical behavioral and treatment interventions [18]. In another modeling study, the potential impact of the Swiss HCVree Trial was assessed [24]. In this trial, a behavioral intervention that prevents HCV transmission through individual risk counseling was combined with early DAA treatment. The model suggested that treatment plus counseling can reduce HCV prevalence among HIV-positive MSM. These modeling studies indicate that early treatment in combination with the implementation of health promotion interventions that reduce high-risk behavior is an effective strategy to control the HCV epidemic in MSM. Targeted socioculturally acceptable preventive measures, including behavioral interventions for MSM at risk of HCV infection, are scarce and urgently needed.

In 2016, we established the Amsterdam MSM Hepatitis C Free (MC Free) consortium with the goal of stopping HCV transmission among MSM in Amsterdam. MC Free is the driving force behind the NoMoreC project, an innovative project co-created with the Amsterdam gay community. Actively involving the target group in the development and implementation of an intervention is a promising approach to increase engagement of the target population [25]. This asks for strong collaboration between health care professionals, researchers, and the target group. Using a co-creational approach ensures that the design is pragmatic, local, and tailored to the target group and the specific settings for which it is created. Consequently, co-creation results in contextually appropriate intervention strategies as well as creation of a platform for co-learning and enhancing ownership and empowerment among the target group [26,27].

For the NoMoreC project, a multilevel intervention was developed and implemented at the individual, community, health care professional, context, patient, and network levels. We believe that targeting these levels simultaneously will lead to a more substantial impact on risk reduction behavior, testing frequency, early diagnosis, early treatment, and partner notification than a single-level intervention would. In this paper, we describe the co-creation process including the development and implementation of the various components of the multilevel intervention.

## Methods

### MC Free Consortium

In January 2016, the Public Health Service of Amsterdam (PHSA), Amsterdam University Medical Centers, location Academic Medical Center, Soa Aids Nederland, and Amsterdam Institute of Global Health partnered to establish the Amsterdam MSM Hepatitis C (MC Free) consortium. Soa Aids Nederland is a nongovernmental organization specializing in sexual health and sexually transmitted infections (STIs) with strong links with the gay community. MC Free is advised by an international scientific advisory board and a local community advisory board. The consortium combines the knowledge and expertise of professionals from different backgrounds and members of the gay community to work towards a common goal: the elimination of HCV among MSM in Amsterdam.

### Multilevel Intervention

MC Free developed the NoMoreC project, a multilevel intervention strategy targeting 6 levels: individual, community, professional, context, patient, and network. For each level, we formulated specific aims (Table 1).

**Table 1:** Levels and corresponding project aims and target groups of the NoMoreC project.

Level	Aims	Target group or context
Individual level	Increase HCV^a^ knowledge and awareness, promote regular testing, and promote risk reduction behavior	MSM^b^ at risk of contracting hepatitis C, including MSM with high-risk sexual behavior and MSM who have sex in networks where hepatitis C infections occur
Community level	Increase HCV knowledge and awareness, promote regular testing, promote risk reduction behavior, and create an atmosphere where men feel responsible for keeping their community HCV-free	Gay community
Professional level	Increase HCV knowledge and awareness, increase knowledge about the available HCV testing options, increase knowledge about risk-reduction strategies, and improve partner notification	Health care professionals who provide care for MSM at risk of hepatitis C, including professionals at the PHSA^c^ STI^d^ clinic, professionals at HIV treatment centers, and GPs^e^ with a substantial number of MSM attending their practices
Context level	Create an enabling environment for risk reduction^f^	Sex on premises venues and organizers of sex parties
Patient level	Provide fast linkage to care and prevent reinfection	MSM who acquired HCV
Network level	Penetrate social and sexual networks where hepatitis C infections occur and enhance partner notification	Social and sexual networks where hepatitis C infections occur

^a^HCV: hepatitic C virus.

^b^MSM: men who have sex with men.

^c^PHSA: Public Health Service of Amsterdam.

^d^STI: sexually transmitted infection.

^e^GPs: general practitioners.

^f^An enabling environment is an environment where risk reduction is facilitated and products for risk reduction are available.

### Co-Creation Process

**Figure 1:**
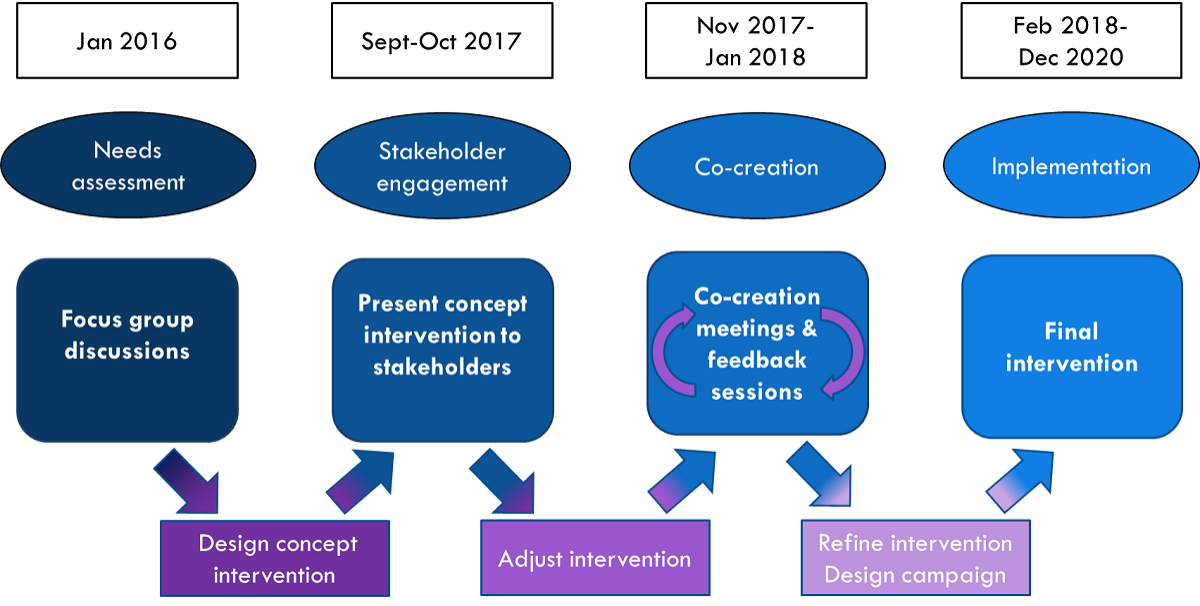
Flow chart of the co-creation process leading to the implementation of the final intervention.

The NoMoreC project was developed in close cooperation with health professionals and the gay community in Amsterdam. Commercial stakeholders and stakeholders from within the gay community were involved at an early stage. The process had 4 distinct phases: a needs assessment, stakeholder engagement, co-creation, and implementation phase (Figure 1).

#### Needs Assessment

In January 2016, prior to development of the intervention, we conducted 2 focus group discussions to identify and discuss the needs regarding hepatitis C information and testing options. MSM at risk of HCV infection were recruited through MSM cohorts of the PHSA, 4 HIV treatment centers in Amsterdam, the Dutch HIV patient association, a magazine for people living with HIV, and a gay dating site. One focus group was held with 7 HIV-positive MSM who had been HCV-infected in the past and 1 HIV-positive MSM who was under HCV treatment at the time of the focus group discussion. The participants of the second focus group were HIV-positive MSM who had never been HCV-infected but had concerns about becoming infected. The following themes were discussed in both focus groups: hepatitis C information (need and current availability), hepatitis C risk, concerns of getting (re)infected, desired hepatitis C prevention tools, motivators and barriers for risk reduction strategies during sex, and different hepatitis C testing options. Structured questions were formulated and posed to the groups, touching upon all themes. The themes were expanded upon depending on the group dynamics. Responses were categorized and clustered per theme.

In addition, 3 intervention ideas were suggested by the researchers: (1) home-based HCV testing service, (2) checklist to estimate personal risk of contracting hepatitis C, and (3) toolbox containing items to assist in HCV risk reduction. The feedback and opinions of the participants regarding these suggestions were collected and processed later in the further development of these interventional aspects.

#### Stakeholder Engagement

Based on the needs assessment, a first concept of the NoMoreC multilevel intervention was designed and presented to a group of commercial and gay community stakeholders in September 2017. This group consisted of owners or managers of sex on premises venues (SOPV), gay chat and dating sites, fetish shops, (sex) party organizers, and representatives of HIV interest groups and organizations active in the gay or HIV community. In October 2017, we organized a second stakeholder meeting for professionals who either treat HCV-infected individuals or are involved in the prevention and control of HCV infections. The meeting was attended by nurses, nurse practitioners, and physicians from HIV treatment centers and STI clinics in Amsterdam; policy advisors and program managers from the National Institute for Public Health and Environment; and Soa Aids Nederland. A total of 9 commercial stakeholders, 4 gay community stakeholders, and 31 professionals attended the meetings. Furthermore, presentations were organized for the clinical staff of HIV treatment centers in Amsterdam. The goal of the stakeholder meetings and presentations was to introduce the NoMoreC intervention and ask for input on the development and implementation of the project as well as to strengthen relationships with stakeholders. Input given during the meetings was used to adjust the concept NoMoreC intervention.

#### Co-Creation Phase

The first co-creation meeting with members of the gay community (n=11) to shape the NoMoreC project was organized in July 2017 prior to the stakeholder meetings, where the first concept intervention was presented. An intensive co-creation phase followed from November 2017 until February 2018 with a group of men from the Amsterdam gay community at risk of HCV or men who had been HCV-infected. The co-creation phase was an iterative process with co-creation meetings and feedback sessions that were used to constantly refine the intervention. During this phase, the group brainstormed, and ideas were discussed and incorporated in the project. Feedback sessions were used to test if the ideas were translated correctly and adjusted where necessary. A total of 78 men contributed to the development of NoMoreC during one or more of the co-creation sessions. Co-creation meetings were held on the development of a hepatitis C prevention toolbox, the online campaign, and outreach activities. The starting point of the meeting about the prevention toolbox was the information collected during the needs assessment. The contents of the NoMoreC toolbox were discussed extensively and decided jointly by the community members and researchers. During the meetings about the campaign and outreach activities, calls-to-action, tone of voice, prevention messages, and different promotion strategies were discussed. Ideas on how to engage men during outreach were proposed and materialized by community members.

## Results

### Co-Creation Process

#### Needs Assessment

The needs and recommendations that were mentioned during the 2 focus group discussions with MSM at risk of HCV are summarized in Table 2.

From the discussions, it became evident that there was a need for clear information about HCV in general and information relevant to the target group and their sexual settings in particular. The participants mentioned that they found it difficult to find reliable information, including information about HCV testing, symptoms, and treatment options. It was mentioned that they felt a good information source was lacking and that a website containing as much information as possible was recommended. They stated that they had a great need for advice regarding their sex lives during the period from the time of infection to cure, including practical tips to limit the risk of transmission during sex. The following quotes illustrate the availability and need for information about HCV.

I use google….but the information is really very limited….there is some information, but it is not a lot.

Participant who had been HCV-infected

…I had questions about that (the risk of transmission during sex), but I now realize that I know little about that.

Participant who had been HCV-infected

I would want to know about the severity of treatment, the duration of the treatment, whether there are treatment choices, what are the prospects (for controlling HCV), what is the chance of a cure, what side effects does it (the treatment) have, what if it (the HCV infection) becomes chronic?

Participant who had never been HCV-infected

….I have had sex with a friend who had HCV. I want to know more about what risk reduction measures you can take to prevent HCV.

Participant who had never been HCV-infected

After discussing HCV information needs, the researchers proposed developing a toolbox containing items to assist in HCV risk reduction and asked the participants’ opinions. The participants reported being interested in a prevention toolbox if it contained disinfectants that quickly kill HCV, clear explanation on its use, and possibly a list of other products that can assist in risk reduction. Furthermore, 2 other intervention ideas were proposed by the researchers: (1) a home-based hepatitis C testing service and (2) an online checklist to estimate personal risk of contracting HCV. We hypothesized that a home-based testing service could lower barriers to testing, increase testing frequency, and enable MSM to test shortly after potential exposure and thus prevent onward transmission. The rationale of the checklist is two-fold: (1) the proposed checklist would guide men in their decision to test for HCV based on their personal risk, and (2) providing tailored practical risk reduction advice may stimulate risk reduction behavior.

These 2 proposed intervention ideas were received positively and discussed by both focus groups and subsequently added as recommendations (Table 2). Offering easier HCV testing options based on measuring the virus within a short period was considered as important by most focus group members, and it was mentioned that this could assist them in taking control of their own health. Some participants were prepared to pay for such a testing service, while others were not and brought up their concern that men with a low income would be excluded from the service.

**Table 2:** Summary results of two focus group discussions determining the needs and recommendations regarding hepatitis C information and testing options.

Theme or topic	Needs	Recommendations
HCV^a^ information	Better HCV information sources	Provide good and reliable online information about HCV, provide information specifically relevant to MSM^b^, link websites visited by MSM to reliable online information about HCV, provide information about symptoms, and provide an online chatroom where men can ask questions to a professional
HCV risk	Information about which sexual techniques confer a transmission risk and personal advice on HCV risk factors	Provide information about which sexual techniques increase the transmission risk and which sexual techniques are safe as well as a risk checklist
HCV prevention	Information about disinfectants effective against HCV and information about sexual behavior that increases HCV risk	Provide information about disinfectants that quickly kill the virus, provide a list with products and how to use these for the prevention of an HCV infection, and create video instructions for risk reduction
HCV testing	Information about different types of tests, information about where you can be tested, and testing possibilities for HIV-negative men outside the general practice	Offer a test that detects the virus (instead of antibodies) that can be used by men who have been infected in the past, offer an HCV test as part of a comprehensive STI^c^ screening package at the PHSA^d^ STI clinic, communicate test results by a telephone call or face-to-face, and offer a home-testing service to take control over your own health (a convenient option and good option for MSM outside of Amsterdam)
A positive HCV test result	Information about disease progression, treatment (duration, side effects, regimens, cure rates), consequences of a chronic infection, safe sex while being infected, and partner notification as well as contact with a gay man who has been infected, treated, and cured	Provide information covering the information needs as mentioned under “needs” in the left adjacent column, offer DAA^e^ treatment as soon as possible, and offer peer support by bringing a newly HCV diagnosed man in contact with a gay man who has had HCV in the past and was treated with DAAs, to share experiences about being infected and the treatment

^a^HCV: hepatitis C virus.

^b^MSM: men who have sex with men.

^c^STI: sexually transmitted infection.

^d^PHSA: public health service Amsterdam.

^e^DAA: direct-acting antiviral.

#### Stakeholder Involvement

Based on the needs and recommendations that were brought forward in the focus group discussions, a first concept of the NoMoreC project was designed and presented to the stakeholders. The project, containing a web-based component, hepatitis C testing service, and face-to-face component, was received with interest. The stakeholder meetings were successful in gathering input and discussing possible support for project implementation. Useful ideas were voiced, and cooperation was offered by various stakeholders: Fetish shop owners offered to distribute or sell potential NoMoreC products as well as assist in project promotion (hand out flyers/posters), and managers of gay sex venues requested to be informed about disinfection on location and made condoms and fisting gloves available for clients, to make their premises “hepatitis C proof.”

#### Co-Creation Phase

During the co-creation phase, different components of the intervention were executed in collaboration with the gay community. These included the development of a prevention toolbox, making content for the project website (eg, explicit comics), filming of testimonials, shooting photos of volunteer models, organization of theme events, and forming a campaign team. The campaign team (the “NoMoreC Boy Scouts”) was formed by a group of men at risk of HCV, including men from specific fetish scenes: leather, rubber, and sportswear [28]. All activities were performed on a voluntary basis. For the coordination of the campaign team, a community member was appointed who received a modest fee. The end result of the co-creation phase was the finalized NoMoreC project including web-based and face-to-face components, an anonymous hepatitis C testing service, and a (social) media campaign. The influence and challenges of the co-creation process are mentioned in the following descriptions of each intervention component.

### Web-Based Intervention Components

#### NoMoreC Website

**Figure 2:**
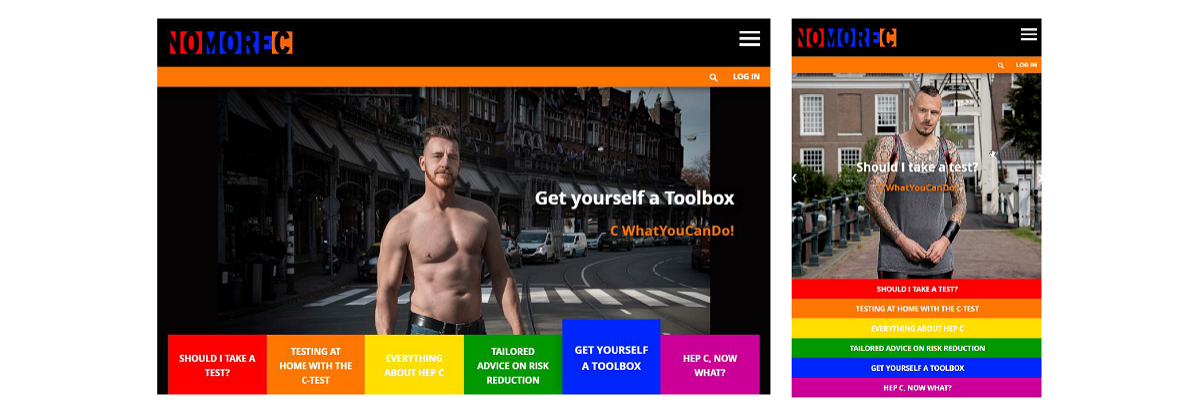
Screenshots of the desktop and mobile versions of the NoMoreC homepage (accessed June 10, 2020).

The project website [29] is available in Dutch and English and targets at-risk MSM (Figure 2). Part of the content was co-created with the community, such as the instructional videos, photos, testimonials, and graphic illustrations. The website was tested by 3 community members. Feedback on their user experience and suggestions for improvement were used to fine-tune the final product. The website was launched in February 2018.

**Figure 3:**
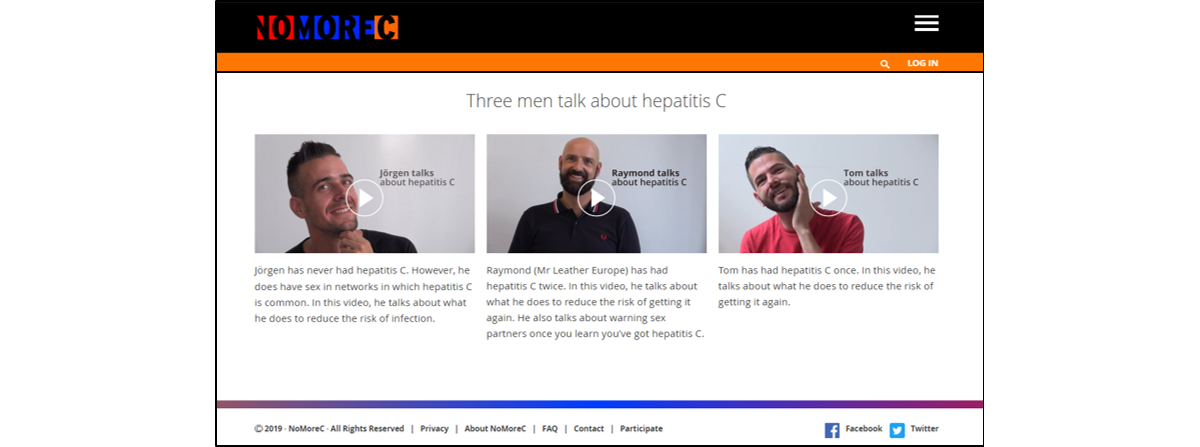
Video testimonials of men from the target population can be watched on the NoMoreC website (accessed June 10, 2020).

The website provides information about hepatitis C, HCV transmission routes, risk reduction strategies, testing and treatment options, and partner notification. Video testimonials are presented in which men share their personal experiences with hepatitis C, being at risk, and what they do to reduce transmission risk (Figure 3).

**Figure 4:**
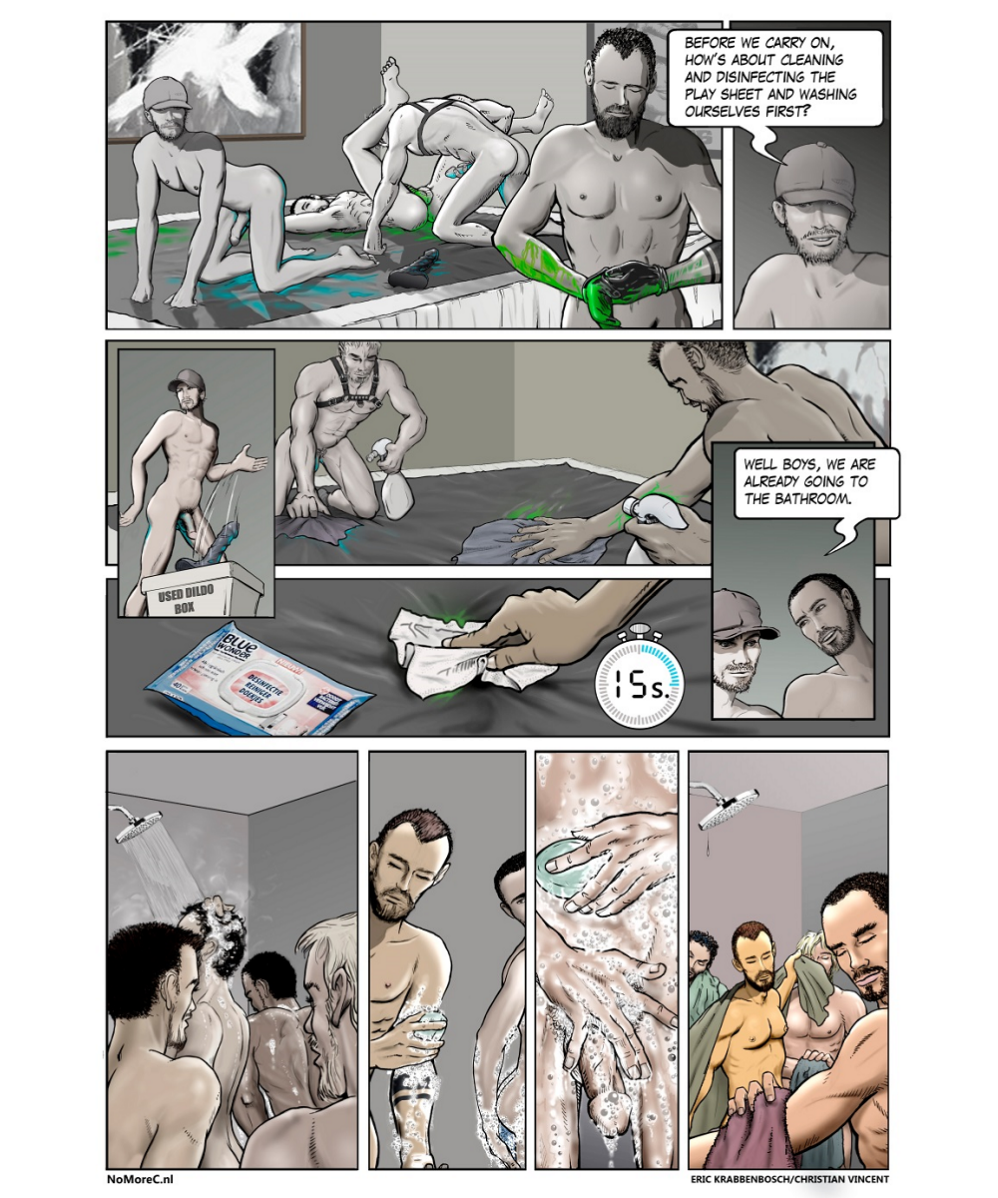
Illustration on the website of a risk reduction strategy: cleaning and disinfection of the play area, toys, and yourself before changing sex partners. More information about the products to use is given on the website.

Information about HCV transmission routes and risk reduction strategies are illustrated by explicit comic strips of situations familiar to the community (Figure 4). Furthermore, instructional videos can be watched about disinfection to prevent HCV transmission. The website offers personalized risk reduction advice based on sexual practices. Also, personalized test advice is given after answering questions on 6 risk factors, based on a previously validated risk score [30]. An anonymous hepatitis C testing service, which is described in detail in a later section, is incorporated in the website. It offers HCV-RNA tests at a reduced cost for €25 per test. A test subscription of 4 tests is offered for €80 to stimulate regular testing.

#### Electronic Learning (e-Learning)

For professionals who see MSM at risk of HCV in their practice, NoMoreC offers an electronic learning (e-learning) package with 3 modules. Module 1 gives information about the NoMoreC project, its target population, and how professionals can use the NoMoreC products in routine clinical practice. Module 2 gives detailed information about HCV risk reduction. Professionals learn about risk factors, tailored risk reduction advice, and behavior change. In the last module, the obligation of health professionals to report hepatitis C to the public health service and partner management is discussed. The e-learning package is accredited by the appointed organizations for nurses and nursing specialists [31,32].

### Anonymous Hepatitis C Testing Service

**Figure 5:**
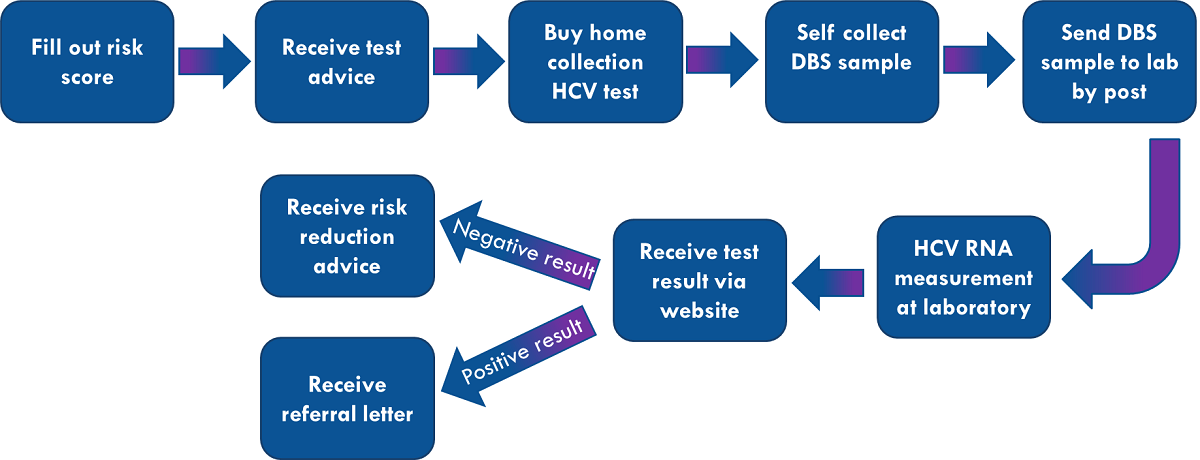
Flow chart of the hepatitis C testing service. DBS: dried blood spot; HCV: hepatitis C virus.

NoMoreC offers an anonymous hepatitis C testing service, using a validated home-based self-sampled dried blood spot (DBS) HCV-RNA test [33]. Users of this service are guided through different steps, as depicted in Figure 5, starting with filling out a validated 6-question risk assessment [30]. After receiving test advice, men can purchase the HCV-RNA home collection test kit online. Test packages are sent to the chosen address of the potential user (pseudonyms can be used). They are instructed to collect a DBS sample from a finger prick, a procedure that was validated prior to offering the test service [33].Paper instructions for DBS collection are included in the test package, and video instructions are accessible on the website. Users are instructed to send their DBS sample to the laboratory of clinical virology of the Amsterdam University Medical Centers for HCV-RNA testing. Test results are communicated within one week via a personal login at the project website. Users who test positive are given guidance on the next step to take, including confirmation of the positive test result at regular health services, access medical treatment and initiate partner notification. A referral letter is provided online to facilitate this follow-up, and a telephone number is given if the user would like to consult a nurse. Post-test information for users who test HCV-RNA negative addresses risk-reduction strategies and encourages frequent testing. The test service became available in February 2018.

During the development of this testing service, we encountered some challenges. Many community members expressed a strong preference towards receiving a positive test result by telephone call or face-to-face, instead of receiving their test result online. However, the possibility of being able to test anonymously was also seen as important. Therefore, the anonymous testing option was chosen with the possibility for men who test positive to be able to contact a nurse and facilitate linkage to care.

### Face-to-Face Intervention Components

#### Risk Reduction Toolbox

**Figure 6:**
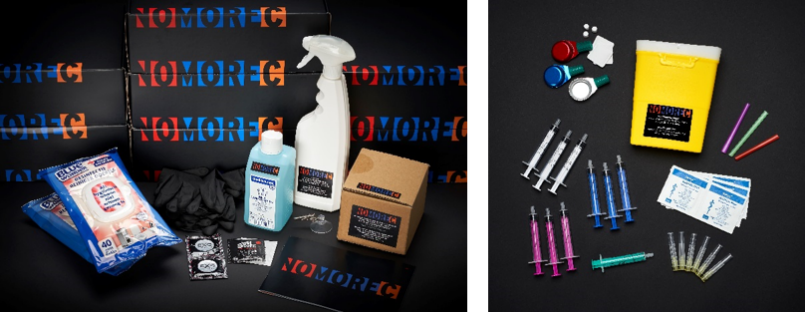
NoMoreC Toolbox (left) and contents of safe drug use box (right). The NoMoreC Toolbox contains a spray bottle, disinfectant for hands, hydrogen peroxide cleaning wipes, gloves, condoms, a hand washing instruction card, a cardboard safe drug use box and booklet with tips and tricks to reduce hepatitis C transmission risk. The safe drug use box is a small cardboard box that is included in the toolbox. It contains snorting straws, a sharps container, syringes, needles, mixing cups, and alcohol wipes. A sticker with the text “Never share drug use equipment. Pick your own color.” is stuck on the box.

The “NoMoreC Toolbox” contains products to assist in reducing the risk of contracting an HCV infection such as condoms, fisting gloves, safe drug use equipment, and disinfectants (Figure 6). We were advised on the right use and types of disinfectants by hygiene and infection prevention specialists of the National Center for Hygiene and Safety [34]. In addition, the toolbox includes a booklet with practical tips on how to reduce risk of transmission and instructions on the use of the products. Information is also given about testing, treatment, and notifying sex partners.

The toolbox is used by health care professionals of the STI clinic and HIV centers in Amsterdam to discuss risk behavior with MSM at risk of HCV and inform them about prevention strategies. The men are offered a box so they can go through it again at home, read the information booklet, and try out the products. The box is also used by the “NoMoreC Boy Scouts” during outreach activities to discuss HCV risk (reduction). Furthermore, the toolbox can be ordered online from the project website or picked up at a gay fetish shop free of charge. The toolbox became available in March 2018.

During the co-creation phase, choices were collectively made on what products the toolbox needed to contain. Consensus was easily reached on the inclusion of the majority of the products except for items for safe drug use. In particular, the suggestion to add needles and syringes to the toolbox led to an extensive discussion. There was disagreement between community members: Some believed that such items would be experienced as shocking while others brought forward that a small group of men inject (“slam”) drugs to enhance their sexual experience and that safe drug use items are essential for the prevention of HCV. A compromise was reached by adding a separate sealed off box to the toolbox, labeled with a sticker explaining that this small cardboard box contains items for safe drug use. After the contents of the toolbox was decided upon, items were ordered, and all boxes were packed by community members. The packing sessions proved to be an effective way to involve men in the project and create a “NoMoreC community.”

#### Training for Health Professionals

The training developed for health professionals is a 4-hour interactive session about HCV risk behavior, risk-reduction measures, and partner management. Prior to the training, participants are asked to complete the e-Learning package. The focus of this training is on improving the participants’ communication skills by practicing motivational interview techniques. After the course, a participant is better equipped to ask a client about his risk behavior, give him tailored advice on risk reduction, and support behavioral change.

#### Tailored Advice to Sex on Premises Venues (SOPV)

In cooperation with infection prevention specialists of PHSA, we advise SOPVs in Amsterdam on HCV prevention and creating an enabling environment for risk reduction for their clients. SOPVs are commercial venues, such as gay clubs and saunas but not brothels, where men can engage in sex. The venues are visited to have a close look at the areas where clients have sex. For each location, tailored advice is given with recommendations for improvement. Points of improvement are given on the use of disinfectants, instructions for cleaning personnel, availability of gloves, single use anal douches, and safe needle disposal. The recommendations are discussed with the owner or manager of the venue 2 months after the visit, giving them the opportunity to ask for clarification. In addition, a workshop for owners of SOPVs is organized, and support is given to venues when requested.

### NoMoreC Campaign

The NoMoreC intervention is promoted with a sex-positive campaign, accepting lifestyle choices of MSM at risk of HCV. The campaign was designed in close cooperation with the community. The goal of the campaign is to raise awareness about the different components of the project that target MSM. With the input of community members, the right words and promotion messages were carefully chosen so that they would resonate with the community. Special care was taken to ensure the messages would neither evoke fear nor stigmatize the target population. The campaign slogan “CWhatYouCanDo!” was chosen to encourage MSM, including men who do not use condoms, to think about which risk reduction strategies they can and are willing to apply to their (sex) lives.

**Figure 7:**
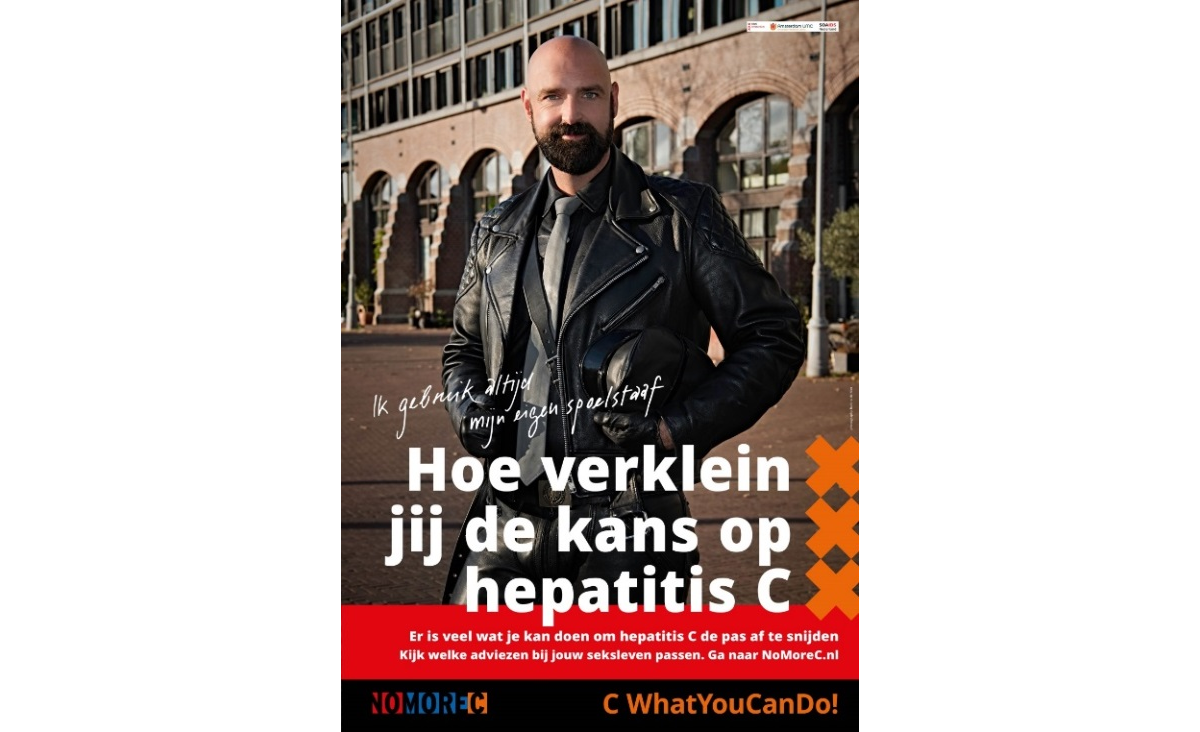
Promotional poster with the text: How do you reduce your hepatitis C Risk “I always use my own anal douche.” The model is a key figure from the target population.

A suite of promotional materials was developed with artwork appealing to the community. It includes posters (Figure 7), flyers, pocket-sized cards, and online banners. Posters hang on waiting room walls of HIV-treatment centers in Amsterdam, the STI clinic of PHSA, and fetish shops. Flyers are handed out to at-risk MSM by health professionals from HIV-treatment centers, the STI clinic, and general practice centers in Amsterdam. Furthermore, at a selection of pharmacies in Amsterdam, flyers are given to men who pick up their HIV medication or pre-exposure prophylaxis. Flyers and pocket-sized cards contain a discount code for the purchase of the NoMoreC HCV-RNA test and are also handed out by the “NoMoreC Boy Scouts” during their outreach activities. This campaign team attends gay venues and events to interact with men at risk of HCV. They have one-on-one discussions about hepatitis C, risk reduction strategies, and testing options; demonstrate the use of the Toolbox products; and host quizzes on HCV and risk reduction.

Banner advertisements are shown on gay (fetish) dating and chat apps, including Recon, Scruff, PlantRomeo, and Grindr, prompting men to visit the NoMoreC website, purchase an HCV-RNA test, or order a toolbox. The placement of the ads is scheduled around gay events (eg, Gay Pride Amsterdam, Folsom Berlin, The Cruise) to raise awareness about possible HCV transmission risks at these events and focus on the importance of testing. Promotional activities will continue until July 2020.

## Discussion

The NoMoreC multilevel intervention was created using a co-creation process involving members of the Amsterdam gay community, commercial stakeholders, stakeholders from within the gay community, and health professionals. This process has resulted in the implementation of web-based and face-to-face interventions, including an informative website, an anonymous HCV testing service, a risk reduction Toolbox, a sex positive campaign, a training package for health professionals, and tailored advice to SOPVs. We believe that co-creation has been one of the main strengths of the project, but it also had its challenges and limitations. Co-creation is a time-consuming and intensive process; it took 2 years from the first focus group discussion to the development of the final intervention. At times, exciting ideas could not be materialized, or a compromise had to be reached that was acceptable for the gay community members, health professionals, and researchers. This required good negotiation and cooperation skills.

To illustrate this, one idea proposed by some members of the gay community was the making of a short film to address hepatitis C risk reduction. In the film, different risk behavior settings and risk reduction strategies would be shown. This would have involved hiring actors and shooting explicit scenes of a group of men at a private sex party, using party drugs, and having sex with multiple partners. There was mixed enthusiasm for this idea. Instead of making a sexually explicit film, it was agreed to make explicit cartoons and instructional disinfection videos (how to disinfect your hands, play area, and sex toys) as an alternative. The cartoonist worked closely with a community member to draw realistic cartoons in a setting recognizable for the target population.

Another challenge was to balance the needs and preferences of the community and data collection needs of the researchers with regard to the HCV testing service. For example, the community members voiced their preference of ordering an HCV test without having to fill out the risk score. However, in order to evaluate the effectiveness of the test service (ie, test advice, orders placed, and test results in relation to the risk taken), this information is needed. Therefore, the wish of the community to order a test without filling out the risk score could not be granted.

The development of the NoMoreC Toolbox has been a positive experience for both the community and researchers. The co-creation sessions created an atmosphere of co-learning, where the researchers learned more about the context of risk behavior and community members became more knowledgeable about hepatitis C, HCV transmission routes, and risk reduction measures.

The NoMoreC project is ongoing and will run until the end of 2020. We continuously monitor the uptake and reflect on the successes and limitations of the intervention and make adjustments if indicated and possible. By trial and error, we find out how to best reach and involve the target group. The uptake of the intervention at the different levels and the acceptability of the NoMoreC project will be evaluated and reported at the end of the implementation phase.

In conclusion, using the process of co-creation, the multilevel NoMoreC intervention was developed and implemented. The intensive cooperation with the community and stakeholders has allowed us to gather their perspectives and incorporate their ideas in the different components of the intervention. The co-creational approach we have taken may serve as a rich and useful source for others who want to develop culturally and context appropriate HCV interventions.

## Abbreviations

DAA: direct-acting antiviral
DBS: dried blood spot
e-Learning: electronic learning
HCV: hepatitis C virus
MC Free: Amsterdam MSM Hepatitis C Free
MSM: men who have sex with men
PHSA: Public Health Service of Amsterdam
SOPV: sex on premises venues
STI: sexually transmitted infection
